# Socioeconomic inequalities in obesity among Korean women aged 19-79 years: the 2016 Korean Study of Women’s Health-Related Issues

**DOI:** 10.4178/epih.e2019005

**Published:** 2019-02-13

**Authors:** Eunji Choi, Ha Na Cho, Da Hea Seo, Boyoung Park, Sohee Park, Juhee Cho, Sue Kim, Yeong-Ran Park, Kui Son Choi, Yumie Rhee

**Affiliations:** 1 Graduate School of Cancer Science and Policy, National Cancer Center, Goyang, Korea; 2 Department of Endocrinology and Metabolism, Inha University School of Medicine, Incheon, Korea; 3 Department of Medicine, Hanyang University College of Medicine, Seoul, Korea; 4 Graduate School of Public Health, Yonsei University, Seoul, Korea; 5 Department of Clinical Research and Evaluation, Sungkyunkwan University, Seoul, Korea; 6 College of Nursing, Yonsei University, Seoul, Korea; 7 Division of Silver Industry, Kangnam University, Yongin, Korea; 8 Department of Internal Medicine, Endocrine Research Institute, Severance Hospital, Yonsei University College of Medicine, Seoul, Korea

**Keywords:** Obesity, Women’s health, Body mass index, Socioeconomic factors

## Abstract

**OBJECTIVES:**

While the prevalence of obesity in Asian women has remained stagnant, studies of socioeconomic inequalities in obesity among Asian women are scarce. This study aimed to examine the recent prevalence of obesity in Korean women aged between 19 years and 79 years and to analyze socioeconomic inequalities in obesity.

**METHODS:**

Data were derived from the 2016 Korean Study of Women’s Health-Related Issues. The chi-square test and logistic regression analysis were used to analyze the associations between socioeconomic factors and obesity using Asian standard body mass index (BMI) categories: low (<18.5 kg/m^2^ ), normal (18.5-22.9 kg/m^2^ ), overweight (23.0-24.9 kg/m^2^ ), and obese (≥25.0 kg/ m^2^ ). As inequality-specific indicators, the slope index of inequality (SII) and relative index of inequality (RII) were calculated, with adjustment for age and self-reported health status.

**RESULTS:**

Korean women were classified into the following BMI categories: underweight (5.3%), normal weight (59.1%), overweight (21.2%), and obese (14.4%). The SII and RII revealed substantial inequalities in obesity in favor of more urbanized women (SII, 4.5; RII, 1.4) and against of women who were highly educated (SII, -16.7; RII, 0.3). Subgroup analysis revealed inequalities in obesity according to household income among younger women and according to urbanization among women aged 65-79 years.

**CONCLUSIONS:**

Clear educational inequalities in obesity existed in Korean women. Reverse inequalities in urbanization were also apparent in older women. Developing strategies to address the multiple observed inequalities in obesity among Korean women may prove essential for effectively reducing the burden of this disease.

## INTRODUCTION

Obesity, which is increasing in prevalence worldwide, is a physical condition characterized by the accumulation of excessive body fat, along with many other health impairments. Obesity has been found to adversely affect a wide spectrum of diseases, from non-communicable disorders to mental and social health, including diabetes, malignancies, depression, discrimination, and others [1-4]. The harmful consequences of being overweight or obese have been found to be especially detrimental in women, elevating risks for reproductive disorders, mental health conditions, and predominantly women’s cancers, such as endometrial and breast cancer [5-8].

Disparities in obesity prevalence have been found to vary according to parameters reflecting differences in socioeconomic status, such as urbanization, education, and income levels. Women residing in urbanized areas and living in socioeconomically-deprived conditions have been reported to show faster increases in the prevalence of obesity and overweight [9-13]. Notwithstanding, the magnitude and direction of effects of socioeconomic factors on inequalities in obesity might vary across countries [14-16]. In North America and European countries, inequalities related to obesity have generally been well documented; obesity has been found to be disproportionately more prevalent among individuals with lower levels of education and income, residents of less urbanized areas, and those lacking access to healthcare [10,15]. In contrast, although only a few studies have addressed socioeconomic inequalities in obesity in developing countries, most findings for these countries indicate contrasting results regarding education and income status (i.e., higher obesity rates in women with higher education and household income) [16]. In light of the results of a prior study that socioeconomic factors affect obesity status in ways that vary by country, it is important to consider how these socioeconomic factors affect obesity to develop health promotion programs.

In the Republic of Korea (hereafter Korea), the prevalence of obesity has been officially surveyed using body mass index (BMI) since 1998. The mean BMI for Korean women slightly increased from 1998 to 2005 and stabilized from 2005 to 2014 [17]. Although the overall prevalence of obesity in women is lower than that of men, Korean women older than 65 years have higher obesity rates than men of a similar age [18]. Additionally, while BMI in Korean men gradually increases with age, in Korean women, it sharply increases after the age of 40 years and menopause [17]. Additionally, despite the stable findings on the overall prevalence of obesity among Korean women, a significant increase in grade II obesity (BMI ≥ 30.0 kg/m^2^) from 1998 to 2014 has been recorded, especially for women aged 20 years to 59 years [17].

While obesity rates in Korea are somewhat lower than those of other Organization for Economic Cooperation and Development countries, socioeconomic inequalities in obesity continue to be a point of discussion [10]. Results from previous studies, however, reflect outdated data [10,19] and provide incomplete conclusions on inequality due to the use of limited inequality indicators [19]. For the most part, those studies adopted conventional logistic analyses or direct simple comparisons of rate differences (excess risk) or ratios between the highest and lowest socioeconomic groups. However, the traditional approach of comparing extreme groups on the socioeconomic spectrum fails to take into account changes across the full range of socioeconomic groups. In this study, we utilized the slope index of inequality (SII) and the relative index of inequality (RII), which are regression-based measures of health status across all ranges of each socioeconomic factor [20]. Furthermore, although previous papers emphasized the discrepancies of obesity patterns by gender, they focused on explaining mechanisms of obesity in Korean adult men due to the higher prevalence of obesity in men [10,19].

Thus, in this study, we aimed to report the prevalence of obesity among Korean adult women aged 19 years to 79 years using recent nationally representative data. We also sought to investigate associations between socioeconomic factors and obesity in Korean women. In particular, we examined existing socioeconomic inequalities in obesity among Korean women according to household income, education level, and urbanization (location of residence) via absolute and relative indicators specialized for inequality analysis.

## MATERIALS AND METHODS

### Study population

Data were acquired from the 2016 Korean Study of Women’s Health-Related Issues (K-Stori) survey. This study was approved by the Institutional Review Board of the National Cancer Center, Korea (approval No. NCC2016-0062). Applying life-cycle-specific questionnaires to women, the K-Stori is a nationwide cross-sectional study designed to investigate a comprehensive range of health conditions in women [21]. A detailed description of K-Stori has been provided elsewhere [21]. In brief, nationally representative participants were selected by multistage random sampling according to population size per region and geographic area, based on Registration Population data for July 2013 from Statistics Korea. Sampling weights were additionally provided to adjust for the age distribution to reflect the total population of Korean women. The survey was conducted from March 1 to May 31, 2016, at which time investigators from a professional research agency went door-to-door to recruit residents, and at least three attempts were made to contact individual residents. The response rate after making contact was 40.4%, excluding those who were absent (24,260) or who did not meet the study criteria (5,691). Each respondent was administered a self-reported questionnaire through an in-person interview. Of the respondents, 9,000 women aged between 19 years and 79 years were finally used for analysis in the current study.

### Measurements

BMI is widely recommended for classifying adults and adolescents as overweight and obese, because of its strong association with health risks, simplicity, and low cost [22]. BMI is calculated as a person’s weight in kilograms divided by his or her height in meters squared (kg/m^2^). The World Health Organization (WHO) advocates standard BMI cut points [22]. However, Asian populations generally tend to have a lower BMI by about 2-3 kg/m^2^; moreover, the WHO standard of BMI classification was not found to correspond to metabolic risk in Asian overweight/obese people [23]. Therefore, the following modified BMI cut points are commonly used for analyses of the Korean population: underweight (<18.5 kg/m^2^), normal weight (18.5-22.9 kg/m^2^), overweight (23.0-24.9 kg/m^2^), and obese (≥25.0 kg/m^2^) [24]. In this study, each respondent provided her self-reported height and weight through the structured questionnaire, which we used to calculate BMI according to the modified Korean version of the WHO classification [17,18]. Based on the BMI classification, the obesity rate was calculated as the number of obese women divided by total number of women.

We analyzed age groups, household income, education level, and urbanization (location of residence) as socioeconomic factors. Considering the women’s life cycle, participants were classified by age into 3 groups to reflect the childbearing period (19-44 years), menopausal period (45-64 years), and older adulthood (65-79 years) [21]. For income, we sequentially classified monthly household income in dollars into 5 groups: 1,999 or below, 2,000-2,999, 3,000-3,999, 4,000-4,999, and 5,000 or above. To facilitate comparisons between our study and previous research in the literature, we utilized categories often adopted in previous studies for classifying education and urbanization [25,26]. Participants were classified by education level into 3 groups: middle school graduate or lower, high school graduate, and college graduate or higher. For urbanization, we first defined metropolitan areas as including 1 special city (Seoul) and 6 metropolitan cities (Busan, Daegu, Incheon, Gwangju, Daejeon, and Ulsan); then, of the remaining areas, those classified as neighborhoods (*dong*) were defined as urban areas, and all remaining areas were defined as rural (*eup* and *myeon*) [25,26]. Additionally, to adjust for the overall health status of the participants, we used a variable for self-reported health status. We asked participants, “in general, would you say that your health is ____?”, with the following 5 scaled items: (1) excellent, (2) good, (3) fair, (4) poor, and (5) very poor. We then classified the 5 items into 3 categories (‘good’ with (1) excellent and (2) good, ‘fair’ with (3) fair, and ‘bad’ with (4) poor and (5) very poor) to include this item in the statistical analysis as a simple and intuitive adjustment factor [27].

### Statistical analysis

The study population was characterized using descriptive statistics. BMI status and socioeconomic factors were presented in tabular form, and the chi-square test was conducted to compare the distributions of the variables. To investigate the associations of socioeconomic factors with obesity, we conducted a logistic regression analysis by the obesity rate. The p-values <0.05 were considered to indicate statistical significance in the chi-square and logistic analyses.

To assess inequalities in obesity, we calculated the SII and the RII for our primary measures. Although analyses of SII and RII require that socioeconomic variables have at least 3 categories and be ordered, they are recommended because they are easy to calculate and interpret [28-30]. The mean values for a health status—here, the mean proportion of being obese (obesity rate)— for each level of a socioeconomic factor are regressed by the midpoints of a distribution of grouped data. Therefore, the strength of these inequality indicators is that they consider not only health states between the extreme ends of a socioeconomic factor, but also all health states for each level of a socioeconomic factor. The SII is interpreted as the absolute difference in health between the theoretically most privileged and the least privileged women. Correspondingly, the RII is obtained by dividing the health status of the most privileged women by that of the least privileged. As relative disparities provide an indication of progress and absolute disparities provide a context for public health interventions, presenting both measures (SII and RII) is essential for deriving complete conclusions [28]. Both were calculated through Poisson regression including age and self-reported health as control variables, because those variables are related with BMI status. By adjusting for age, self-reported health status, and sampling weight in the regression, we tried to estimate the effects of socioeconomic factors on obesity. All statistical analyses were performed using Stata version 13 (StataCorp., College Station, TX, USA).

## RESULTS

The socio-demographic characteristics of the study population are described in Table 1. Overall, 21.2% and 14.4% of Korean women aged 19-79 years in this study were overweight or obese, respectively. In the current study, the majority of women had a college or higher education (44.2%), and resided within a metropolitan area (46.2%). Women aged between 65 years and 79 years comprised the highest proportion of overweight and obese women, followed by women aged between 45 years and 64 years. In contrast, the proportion of underweight was highest among women aged between 19 years and 44 years (Table 1). Women in the lowest class of household income or who had only completed a middle school education or lower also tended to have a higher BMI (Table 1).

The associations between socioeconomic factors and obesity were analyzed using logistic regression by applying weights for age distribution (Table 2). Women aged 45-64 years and 65-79 years were more likely to be obese, as represented by odds ratios (ORs) of 2.35 and 2.93, compared to the youngest age group as a reference group. By household income, only women with the highest level of income were significantly less likely to be obese, compared to women with the lowest income (Table 2). Interestingly, while women with higher levels of education were less likely to be obese, living in metropolitan areas showed a positive association with obesity. Linear trends in the obtained ORs for each socioeconomic variable were estimated and demonstrated consistent and significant increasing or decreasing trends among all socioeconomic factors.

In all women, the SII and RII estimates of inequalities according to education level and urbanization of residence were significant. As the SII refers to the difference in health from the most privileged to the least privileged, positive SII values imply a higher percentage of obesity in women of higher socioeconomic status. Meanwhile, RII reflects the ratio of health between the highest and the lowest socioeconomic groups, indicating fold changes in health between the highest and lowest socioeconomic groups. In our data, the negative SII values for education (-16.66) indicated that obese women had disproportionately less education, by as much as about 16%, while the RII estimate of 0.32 revealed that women with a higher education status had a 0.32 times lower obesity prevalence compared to women with less education (Table 3). Furthermore, as shown in Table 3, women living in metropolitan areas were significantly more likely to be obese than women in rural areas (SII, 4.46; RII, 1.36).

As the prevalence and health effects of obesity differ considerably among age groups and women in different life cycles, we conducted a subgroup analysis according to those categories, as presented in Table 4. Educational inequalities were significant in all age groups, with fewer highly educated women being obese. Younger women, between 19 years and 44 years, showed significant inequalities in obesity according to household income levels (SII, -3.89; RII, 0.49) (Table 4). The SII and RII values for urbanization among women of ages 65 years or over reflected a higher proportion of obesity in women residing in metropolitan areas (SII, 9.65; RII, 1.44) (Table 4).

## DISCUSSION

In the current study, we presented up-to-date findings on the prevalence of obesity among the general population of women in Korea. Although researchers have regularly stressed the importance and existence of socioeconomic inequalities in the Korean population, few studies have investigated socioeconomic inequalities in Asian women with specialized indicators of disparity and the latest data sources. Herein, we discovered that the prevalence of overweight/obesity among Korean women remained steady at about 40%, which is slightly lower than the prevalence of 45.3% reported by Shin et al. [17]. In comparison, the prevalence of overweight/obesity in Korean women was far below that of women from the USA [31], similar to that of Central Asian women, and higher than that of women from most Southeast Asian countries [16,32]. In particular, higher proportions of obesity were recorded for women with the least education and the lowest household income. We also noted significant associations between socioeconomic factors and obesity. Overall, our findings agree with other studies from developed countries, demonstrating that socioeconomically-deprived women suffer from being overweight and obese disproportionately to their counterparts of higher socioeconomic status [16,29,33].

In most developed countries, inverse associations between educational inequalities and obesity status have been detected, which we also observed in Korean women across all age group [34]. The reverse relationship between education attainment and obesity status is generally described as a common phenomenon in the early stages of economic development [35]. In countries such as the UK, where obesity rates have reached “ceiling levels,” educational inequalities are considered insignificant [34]. For the Korea, although the prevalence of obesity in Korean women has stabilized, we discovered significant inequalities in obesity according to education level [19]. Our results are supported by a recent study from the Korea that detected increasing educational inequalities over time from 1998 to 2007 in BMI and waist circumference among women aged 25-64 years [19].

Young women are generally more vulnerable to socioeconomic inequalities in obesity [34]. Women of younger age and with low income tend to have lower self-esteem, which is a factor that influences the risk of becoming obese. Indeed, inequalities according to household income and urbanization differed with age in the present study. Income inequalities were significant in younger women aged 19-44 years, while urbanized residence inequalities were detected in older women aged 65 years and older. Furthermore, the prevalence of obesity among Korean women sharply increased after menopause. Despite a similar trend of increases in overweight and obesity for women aged 45-64 years, no additional socioeconomic inequalities were identified [16,34,36].

Throughout the world, women living in metropolitan areas have been found to be more likely to be obese, in part because of a sedentary lifestyle in urban areas [16,34]. Thus, we further analyzed sedentary behaviors among the women in the oldest age group by using the item “How many hours on average do you spend sitting or lying down from when you wake up until you go to bed?” [37-39]. The mean amount of sedentary hours was highest in women living in a metropolitan area, compared to women in urban and rural areas, with statistical significance based on the *t*-test. Furthermore, upon dichotomizing sedentary hours as ≤5 hours and >5 hours, we discovered that significantly more women residing in metropolitan areas were sedentary for >5 hours per day. Therefore, a sedentary lifestyle might account for the inequalities in obesity from urbanization among older Korean women. Altogether, specialized, tailored support for women of different ages may be needed to address inequalities in obesity among Korean women.

Our study has a number of limitations. First, the cross-sectional design may hinder the ability to implicate any causal relationships underlying the observed associations between obesity and socioeconomic inequalities. Thus, future studies with a longitudinal design are recommended to track changing patterns in health inequalities in obesity among Korean women. In fact, some studies have described decreasing inequalities as the prevalence of obesity increases, while others have suggested that the magnitude of socioeconomic inequalities change only a little, despite very high obesity rates. Findings specific to Korea regarding trends in inequalities in obesity are urgently needed.

Second, we administered a self-reported survey to the participants, possibly causing recall bias or incorrect measures of weight and height. Generally, women are more likely to under-report their weight and over-report their height; however, we did not correct values of weight and height in the analysis. Therefore, we may have underestimated the prevalence of obesity. Moreover, Korean women suffer from not only being obese, but also from several problems related to underweight. However, we made no effort to account for possible effects of these issues on our results. Finally, we derived the analytic data from the K-Stori survey, which was designed to be representative of women in the general Korean population, accounting for age distribution and regional characteristics. Thus, caution is required when generalizing and interpreting our results for other Asian countries.

Despite its limitations, this study is important in that we attempted to examine nationwide socioeconomic inequalities in obesity among Korean women over a wide age range. Through logistic regression, our results demonstrated that economically-deprived and educationally-deprived women were more likely to be obese. Inequality-specialized indicators further revealed significant socioeconomic inequalities among specific age groups of women. As well, this study highlights practical issues that have remained hidden due to the relatively low overall prevalence of obesity in Korean women, providing novel insights of use in developing interventions to address health inequalities at the population level. Tailored strategies to diminish socioeconomic inequalities in obesity among Korean women may play an important role in achieving greater equity and efficacy in health care programs.

## Figures and Tables

**Table 1.** Baseline characteristics of the study population in Korea according to body mass index (BMI)

		Total	BMI (kg/m^2^)				p-value
			Underweight (<18.5)	Normal weight (18.5-22.9)	Overweight (23.0-24.9)	Obese (≥25.0)	
Total		9,000 (100)	474 (5.3)	5,320 (59.1)	1,909 (21.2)	1,297 (14.4)	
Age (yr)	19-44	4,118 (45.7)	380 (9.2)	3,009 (73.1)	497 (12.1)	232 (5.6)	<0.001
	45-64	3,554 (39.5)	69 (1.9)	1,813 (51.0)	1,027 (28.9)	645 (18.1)	
	65-79	1,328 (14.7)	25 (1.9)	497 (37.4)	385 (30.0)	420 (31.6)	
Self-reported health status	Bad	913 (10.1)	15 (1.6)	416 (45.6)	236 (25.8)	246 (26.9)	<0.001
	Normal	2,772 (30.8)	125 (4.5)	1,464 (52.8)	672 (24.2)	511 (18.4)	
	Good	5,315 (59.1)	334 (6.3)	3,440 (64.7)	1,001 (18.8)	540 (10.2)	
Monthly house-hold income (US$)	1,999 or below	1,238 (13.8)	35 (2.8)	534 (43.1)	330 (26.7)	340 (27.5)	<0.001
	2,000-2,999	1,367 (15.2)	48 (3.5)	706 (51.6)	354 (25.9)	258 (18.9)	
	3,000-3,999	2,387 (26.5)	127 (5.3)	1,457 (61.0)	526 (22.0)	276 (11.6)	
	4,000-4,999	1,941 (21.6)	95 (4.9)	1,233 (63.5)	377 (19.4)	236 (12.2)	
	5,000 or above	2,069 (24.0)	169 (8.2)	1,390 (67.2)	322 (15.6)	187 (9.0)	
Education level	Middle school graduate or lower	1,651 (18.3)	25 (1.5)	621 (37.6)	495 (31.0)	511 (30.9)	<0.001
	High school graduate	3,371 (37.5)	108 (3.2)	1,808 (53.6)	904 (26.8)	551 (16.3)	
	College graduate or higher	3,978 (44.2)	341 (8.6)	2,891 (72.7)	511 (12.8)	235 (5.9)	
Urbanization	Rural area	1,516 (16.8)	84 (5.5)	857 (56.5)	359 (23.7)	216 (14.2)	0.025
	Urban area	3,328 (38.0)	187 (5.6)	2,013 (60.5)	674 (20.2)	453 (13.6)	
	Metropolitan area	4,157 (46.2)	202 (4.9)	2,450 (58.9)	877 (21.1)	628 (15.1)	

Values are presented as number (%).

**Table 2.** Logistic analysis of socioeconomic factors and obesity status in Korea, 2016 (n= 9,000)

	Categories	aOR (95% CI)	p for trend^1^
Age (yr)	19-44	1.00 (reference)	<0.001
	45-64	2.35 (1.96, 2.81)	
	65-79	2.93 (2.25, 3.77)	
Self-reported health status	Bad	1.00 (reference)	<0.001
	Normal	0.90 (0.74, 1.08)	
	Good	0.67 (0.55, 0.81)	
Monthly household income (US$)	1,999 or below	1.00 (reference)	0.041
	2,000-2,999	1.00 (0.81, 1.23)	
	3,000-3,999	0.85 (0.68, 1.05)	
	4,000-4,999	1.01 (0.80, 1.28)	
	5,000 or above	0.77 (0.61, 0.99)	
Education level	Middle school graduate or lower	1.00 (reference)	<0.001
	High school graduate	0.69 (0.57, 0.84)	
	College graduate or higher	0.36 (0.28, 0.46)	
Urbanization	Rural	1.00 (reference)	0.002
	Urban	1.10 (0.92, 1.32)	
	Metropolitan	1.32 (1.10, 1.57)	

aOR, adjusted odds ratio; CI, confidence interval.

1 Significance of linear trend among ORs.

**Table 3.** Socioeconomic inequalities in obesity status among in Korea women, 2016^1^ (n= 9,000)

	SII (95% CI)	RII (95% CI)
Education level	-16.66 (-21.55, -6.09)	0.32 (0.23, 0.45)
Monthly household income	-2.67 (-6.09, 0.74)	0.96 (0.77, 1.19)
Urbanization	4.46 (1.50, 7.42)	1.36 (1.15, 1.61)

SII, slope index of inequality; RII, relative index of inequality; CI, confidence interval.

1 Adjusted for self-reported health status and age.

**Table 4.** Socioeconomic inequalities in obesity status according to age groups in Korea, 2016^1^ (n= 9,000)

Age (yr)	Socioeconomic variables	SII (95% CI)	RII (95% CI)
19-44	Education level	-9.78 (-14.62, -4.95)	0.21 (0.10 , 0.44)
	Monthly household income	-3.89 (-7.62, -0.17)	0.49 (0.26, 0.92)
	Urbanization	1.48 (-2.27, 5.23)	1.16 (0.69, 1.95)
45-64	Education level	-17.34 (-23.99, -10.69)	0.37 (0.23, 0.59)
	Monthly household income	2.56 (-3.36, 8.47)	1.12 (0.78, 1.60)
	Urbanization	2.94 (-2.42, 8.30)	1.27 (0.94, 1.70)
65-79	Education level	-10.93 (-21.22, -0.64)	0.57 (0.30, 0.98)
	Monthly household income	-4.13 (-11.82, 3.55)	0.80 (0.58, 1.11)
	Urbanization	9.65 (2.83, 16.47)	1.44 (1.15, 1.81)

SII, slope index of inequality; RII, relative index of inequality; CI, confidence interval.

1 Adjusted for self-reported health status and age.
